# Inner Ear and Muscle Developmental Defects in Smpx-Deficient Zebrafish Embryos

**DOI:** 10.3390/ijms22126497

**Published:** 2021-06-17

**Authors:** Anna Ghilardi, Alberto Diana, Renato Bacchetta, Nadia Santo, Miriam Ascagni, Laura Prosperi, Luca Del Giacco

**Affiliations:** 1 Department of Biosciences, Università degli Studi di Milano, Via Celoria 26, 20133 Milan, Italy; anna.ghilardi@unimi.it (A.G.); alberto.diana@unimi.it (A.D.); lau.prosperi@gmail.com (L.P.); 2 Department of Environmental Science and Policy, Università degli Studi di Milano, 20133 Milan, Italy; renato.bacchetta@unimi.it; 3 Unitech NOLIMITS, Università degli Studi di Milano, 20133 Milan, Italy; nadia.santo@unimi.it (N.S.); miriam.ascagni@unimi.it (M.A.)

**Keywords:** zebrafish, *SMPX*, hearing loss, myopathy, X-chromosome

## Abstract

The last decade has witnessed the identification of several families affected by hereditary non-syndromic hearing loss (NSHL) caused by mutations in the *SMPX* gene and the loss of function has been suggested as the underlying mechanism. In the attempt to confirm this hypothesis we generated an Smpx-deficient zebrafish model, pointing out its crucial role in proper inner ear development. Indeed, a marked decrease in the number of kinocilia together with structural alterations of the stereocilia and the kinocilium itself in the hair cells of the inner ear were observed. We also report the impairment of the mechanotransduction by the hair cells, making *SMPX* a potential key player in the construction of the machinery necessary for sound detection. This wealth of evidence provides the first possible explanation for hearing loss in *SMPX*-mutated patients. Additionally, we observed a clear muscular phenotype consisting of the defective organization and functioning of muscle fibers, strongly suggesting a potential role for the protein in the development of muscle fibers. This piece of evidence highlights the need for more in-depth analyses in search for possible correlations between *SMPX* mutations and muscular disorders in humans, thus potentially turning this non-syndromic hearing loss-associated gene into the genetic cause of dysfunctions characterized by more than one symptom, making *SMPX* a novel syndromic gene.

## 1. Introduction

Perhaps the most common sensory human condition, hearing loss concerns roughly 5% of the world population. Fifty percent of the cases can be genetically explained and more than 35% of this subgroup are classified as non-syndromic (non-syndromic hearing loss, NSHL) ([1] and references therein). The rarest, contributing to less than 1% of the total cases of NSHL, is the X-linked form.

The evolutionarily-conserved cytoskeleton-associated protein SMPX encoding gene, located on the mammalian X-chromosome, is highly expressed in the hair cells of the vertebrate inner ear during development from zebrafish to humans [2,3,4]. Specifically, as we revealed in zebrafish, SMPX is embedded in the cuticular plate (CP) of the hair cell, an actin-based specialized structure located underneath the apical plasma membrane [4], which profoundly influences mechanotransduction or the ability of hair cells to convert a mechanical stimulus (*i.e.*, an acoustic wave) into a neural signal ([5] and references therein). Indeed, the CP is the anchoring region for the actin-containing rootlets of the stereocilia, which, in turn, form the sensory bundle of the vertebrate inner ear hair cells that bends under mechanical stimuli. CP has been proposed as critical in conferring the bundle its resistance to the continuous stress imposed by mechanotransduction [6].

Interestingly, *SMPX* is heavily expressed in muscles, another tissue that depends on mechanotransduction [2,3,4], and localizes at focal adhesions, interacting with other focal adhesions proteins such as vinculin [7], which are complexes behaving as miniaturized mechanosensors connecting cells to their microenvironment in a dynamic fashion [8]. It has been also shown that SMPX participates in the regulation of the dynamics of the cytoskeleton through the modulation of the Rac1/p38 pathway [7] and coordinates the state of muscle cells, enhancing the NFAT and MEF2 transcription factor activity via the interaction with the IGF-1 signaling [9].

Noteworthy, all of these players are also involved, at some level, in ear development and human deafness [10,11,12,13,14,15]. Finally, the RFX transcription factors, which are crucial for hearing in mice, and the Rbm24 RNA-binding protein, which is expressed in the mechanosensory cells of the auditory/vestibular system during vertebrate development, positively regulate *Smpx* expression [16,17].

Mutations in *SMPX* represent the genetic origin to explain several cases of X-linked congenital deafness and progressive NSHL in humans [1,2,3,18,19,20,21,22,23,24,25]. All of the pathogenic forms of *SMPX* are associated with the transcription and maturation of aberrant mRNAs that face degradation through the nonsense-mediated mRNA decay process. Therefore, the loss of function has been suggested to be the mechanism triggering hearing impairment in SMPX patients [18]. However, the effects of *SMPX* misregulation on otic development have never been investigated in all in-vivo models generated until now [9,26]. In this study, we employ zebrafish to mimic the lack of functional SMPX, representative of the condition in the inner ear hair cells of patients, through the downregulation of *smpx* by means of two gene-specific Morpholino antisense oligomers. Using the Smpx-deficient larvae, we show that Smpx plays a critical role in the organization of the sensory bundle of hair cells: a low level of Smpx is associated with aberrant stereocilia and a profound alteration of the kinocilium; the latter is involved in the morphogenesis of the hair bundle itself [27,28]. Finally, we demonstrate that *smpx* downregulation hampers the capability of hair cells to perform mechanotransduction.

These results reveal the crucial function of SMPX in the morphogenesis and functioning of the inner ear hair cells of vertebrates and provide the first potential functional link between the hearing loss of patients and *SMPX* mutations.

Despite its significant expression in the skeletal muscle of all vertebrate species analyzed, *Smpx* knock-out or overexpression in rodents did not result in any noticeable muscular phenotype, nor have *SMPX* mutations been associated with muscular alterations in patients [9]. Nevertheless, in contrast to this previous evidence, we show here that zebrafish Smpx-deficient larvae display a clear impairment in proper muscle development with severe defects in the organization of muscle fibers, which also affect the larvae’s movements. Therefore, this last surprisingly result makes it worth investigating possible correlations between *SMPX* mutations and muscular disorders in humans.

On the other hand, in agreement with previous studies, our data reveal that Smpx overexpression is not associated with any gross changes in all zebrafish territories analyzed; namely, the inner ear and muscle fibers.

Finally, using an Smpx-GFP chimera, we describe the subcellular localization of the protein in muscle fibers during development.

## 2. Results

### 2.1. Morpholino-Mediated smpx Knockdown

MO1 and MO2 oligomers, both targeting two different sequences flanking the ATG region of the *smpx* mRNA, were employed to functionally ablate the gene in a specific fashion as we confirmed by rescuing the effects caused by the MOs co-injecting *smpx* synthetic mRNA properly mutagenized in the region targeted by the MOs (data available upon request). Additionally, for the in-vivo test of the specificity of the gene-specific two MOs, an N-term Smpx:GFP sensor was generated (Figure S1).

All of the phenotypes described thereafter were obtained with each one of the MOs used independently.

### 2.2. Morphant Larvae Are Characterized by a Curly Body Axis

**Figure 1:**
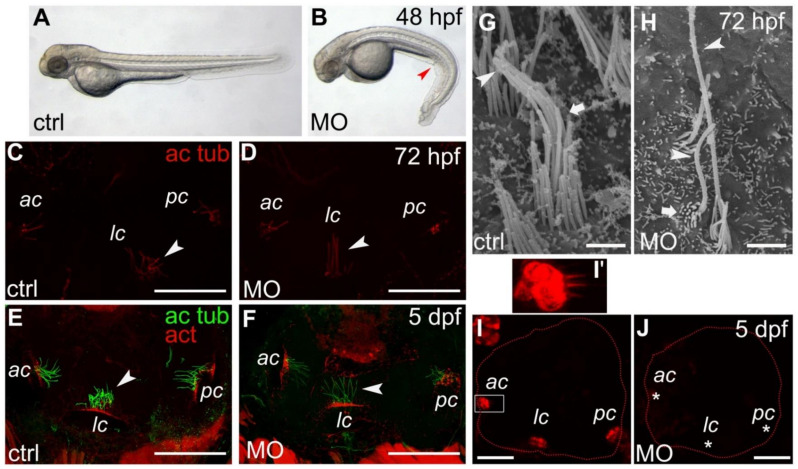
Phenotypical and ear defects in *smpx* zebrafish morphants (MO). (**A**,**B**) representative phenotype caused by the lack of Smpx. Control-MO (**A**) and smpx-MO (**B**) injected embryos. The red arrow points to the downward trunk/tail curvature indicative of cilia disfunction. (**C**–**F**) kinocilia of the anterior (ac), lateral (lc) and posterior (pc) *cristae* of the inner ear of 72 hpf (**C**,**D**) and 5 dpf (**E**,**F**) larvae injected with ctrl- (**C**,**E**) and smpx-MO (**D**,**F**). *Cristae* kinocilia are stained with the antibody against acetylated tubulin (ac tub); phalloidin is used to label the actin (act) of the stereocilia. White arrowheads indicate the different ‘posture’ of the lateral *crista* kinocilia. (**G**,**H**) SEM images of the ciliary bundle in the control larvae (**G**) and *smpx* morphants (**H**). The arrowhead indicates the kinocilium, with the arrow the stereocilia. (**I**,**J**) representative confocal images of the otic cavity injected with FM4-64, labeling the inner ear hair cells in the control larvae ((**I**), n = 4) and *smpx* morphants ((**J**), n = 7). The asterisks indicate the lack of signal in the ac, lc and pc of the Smpx-deficient larvae, suggestive of an impaired mechanotransduction. (**I’**) magnified view of the anterior *crista* in (**I**) (white rectangle). Scale bars = 50 μm in (**C**–**F**); 25 μm in (**I**,**J**); 1 μm in (**G**,**H**).

According to previous published papers, the so-called zebrafish embryo ‘cilia mutants’ as well as morphants are marked by a profound downward trunk/tail curvature, which represents a phenotypic readout for cilia disfunction [29,30]. Quite interestingly, our Smpx-deficient larvae showed this distinctive signature (Figure 1A,B) in 80% of the cases (n = 350), strongly supporting the involvement of *smpx* in proper cilia development/functioning.

### 2.3. Apical Hair Bundle Alteration in *Smpx*-Deficient Larvae

Inner ear sensory hair cells transduce mechanical stimuli by their apical hair bundle, the latter comprising the kinocilium and several stereocilia. Although the kinocilium was initially supposed not to be part of the mechanotransduction phenomenon, it basically is involved in directing the correct assembly of the bundle; more recent results uncovered its main role in mechanosensation during zebrafish development [31].

Following MO injections, we found that the gross morphology of the otic vesicle was normal as also shown by the expression of the ear-specific markers *fgf8* (Figure S2A,B) and *pax5* (Figure S2C,D), as well as F-actin (Figure S2E,F) at two different developmental stages. Nevertheless, a more in-depth investigation conducted by immunofluorescence on whole larvae revealed a distinguishing phenotype characterizing the kinocilia of *maculae* and *cristae*. Indeed, morphant kinocilia were never ‘curly’, as always in the preparations of larvae injected with the control MO, but displayed a ‘straight posture’ in all sensory patches at all stages analyzed (Figure 1C–F, n = 10 for each time point). Although the number of kinocilia appeared lower in the morphant larvae, the straight versus the curly phenotype did not make it possible to perform the exact counting of the kinocilia in each sensory patch, nor did it allow the measurement of their length. Therefore, we analyzed the larvae inner ear by SEM, revealing that, in the absence of Smpx, the apical hair bundle morphology was deeply affected, with the kinocilium showing an alteration in length (both hyper- or hypo-developed) and the stereocilia being dramatically shorter; the latter formed thinner bundles in comparison with the controls (Figure 1G,H and Figure S3).

Finally, we also investigated *smpx* expression following the upregulation of Atoh1, a transcription factor necessary for both the differentiation of hair cells and for the transdifferentiation of supporting cells into hair cells when the latter are depleted [32]. Although Cai and colleagues suggested that *Smpx* expression might be regulated by Atoh1 in P1 mouse inner ear hair cells after RNA-Seq profiling [32], we did not detect any changes in the distribution of *smpx*-specific signals in the inner ear of 48 hpf larvae by whole-mount in-situ hybridization (WISH; Figure S4). The discrepancy might be due to the different sensitivity of the two approaches as well as the stage of development analyzed in the mouse and zebrafish embryos. However, Millimaki and colleagues [33] reported how the overexpression of *atoh1* in zebrafish did not result in an increase in the hair cell number at the stage we analyzed (48 hpf). Therefore, additional analyses are needed to clarify the Atoh1 contribution to *smpx* expression.

### 2.4. Lack of *Smpx* Affects Mechanotransduction in Zebrafish Larvae Inner Ear Hair Cells

The dramatic alteration of both the kinocilium and stereociliary bundle was suggestive of an impairment of mechanotransduction. Therefore, we pressure-injected the FM4-64 vital fluorophore into the Smpx-deficient larval otic cavity and demonstrated the complete lack of mechanotransduction when the protein was absent (Figure 1I,J). Indeed, FM4-64 can permeate through the mechanoelectrical transduction (MET) channels located at the tip of the stereocilia exclusively when they open in the act of depolarizing the hair cell, turning the mechanical stimulus into the electrical impulse traveling to the brain [34]. The complete lack of signal in the Smpx-morphants, demonstrating the absence of hair cell activity, highlighted the importance of the *smpx* contribution to the process of hearing.

### 2.5. *Smpx* Is Critical for the Proper Organization of the Larvae Muscle Fibers

As *smpx* was abundantly expressed in muscle progenitor cells during somitogenesis [4], we next focused our attention to both slow and fast muscle fibers of the morphant larvae. Although all of the ‘curved’ embryos displayed profound alterations of muscle development, the downregulation of *smpx* did not affect the balance between slow and fast fibers, as shown by the expression of their specific markers, *smyhc1* and *myhz2*, respectively. Indeed, the WISH analysis did not evidence any difference in the reciprocal distribution of the two fiber types in comparison with the controls (Figure S5A–D). This result was also supported by the RT-PCR expression analysis of both genes at 24, 48 and 72 hpf, which did not show any significant changes in the mRNA amount during the time course considered (data available upon request).

**Figure 2:**
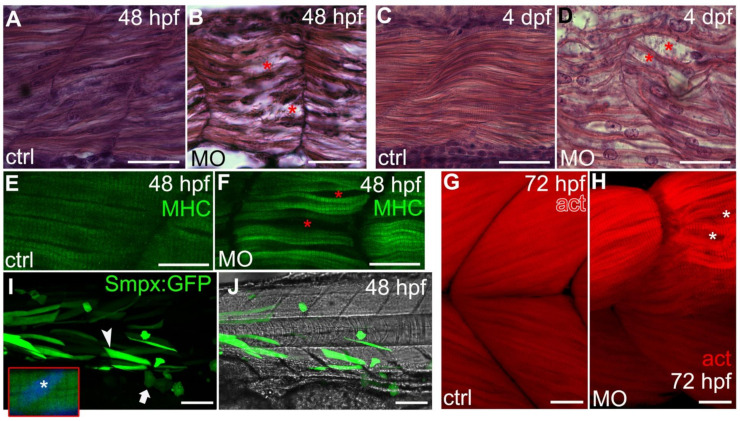
*smpx* knockdown disturbs the proper fiber arrangement during development. (**A**–**D**) side views, anterior left, dorsal top. Paraffin sections of Control-MO (**A**,**C**) and smpx-MO (**B**,**D**) injected embryos at 48 hpf (**A**,**B**) and 4 dpf (**C**,**D**). The red asterisks indicate alterations in the muscle array. (**E**–**H**) side views, anterior left, dorsal top. Confocal Z-stacks taken from whole-mount embryos (48 hpf) and larvae (72 hpf) labelled with (**E**,**F**) myosin heavy chain (MHC) antibody for slow fibers and (**G**,**H**) phalloidin (act) for actin filaments of the fast fibers. The red (**F**) and white (**H**) asterisks indicate alterations in the normal muscle array (controls in (**E**,**G**), respectively). (**I,J**) representative lateral view of an embryo injected with the construct encoding the Smpx:GFP chimera (see Figure S1A for construct details); muscle fibers (arrowhead) and epithelial cells (arrow) are both uniformly painted with no accumulation of Smpx:GFP in the nuclei. Inset: magnification of a slow fiber with the nucleus (asterisk) labelled with DAPI (blue). Anterior left, dorsal top. Scale bars = 25 μm in (**A**–**D**); 20 μm in (**E**,**F**); 20 μm in (**G**,**H**); 100 μm in (**I**,**J**).

Nevertheless, the organization of both types of fiber was severely hampered when Smpx was decreased, resulting in them being clearly disorganized, less dense and compacted (Figure 2 and Figure S5E,F). To evaluate the muscular activity of *smpx* morphant embryos, a touch-evoked response assay was performed. As expected from the general disorganization of the fibers, the downregulation of *smpx* caused the larvae to require a number of stimuli significantly higher than the controls to evoke a startle response (Figure S6), suggestive of an impairment in muscle functioning. Quite interestingly, the potential for the association between *SMPX* mutations and muscle conditions in humans has been very recently reported [35]. Indeed, the authors identified *SMPX* as a novel disease gene responsible for the first form of distal myopathy linked to the X-chromosome.

Reactive oxygen species (ROS) production is one of the main events occurring during muscle contraction and the generation of ROS has been associated with both physiological and pathological muscle damage; the latter including various muscular disorders [36]. As the morphant muscles were clearly affected by the lack of Smpx, we investigated the expression of *sod1* and *sod2*, two gene encoding enzymes that dismutate superoxide radicals to protect muscles from oxidative damage. While the expression of *sod1* was not influenced by Smpx deficiency, the amount of *sod2* mRNA was significantly decreased by two-fold below the control level (Figure S5G,H), thus suggesting that the muscular phenotypes we observed following *smpx* downregulation might be related, at least in part, to ROS accumulation.

### 2.6. *Smpx* Overexpression and Protein Subcellular Localization

We investigated the possible effects of *smpx* upregulation via a synthetic mRNA microinjection on both ear and muscle fibers. The increase in the amount of the protein was not associated with any developmental phenotype (Figure S1B,D,F and data not shown), as also previously reported in rat muscles [26]. Moreover, although we never detected Smpx in the nucleus of the cells by means of protein-specific antibodies ([4] and this manuscript), as previously suggested [37], we chose to deepen our knowledge concerning the subcellular localization of Smpx, which might be critical to the understanding of Smpx biological activities, probing the position of the protein *in vivo* using GFP as a reporter. Therefore, we generated transiently transgenic larvae by the injection of a DNA vector bearing the Smpx coding sequence that we C-terminally fused to the GFP open reading frame under the control of the cytomegalovirus (CMV) promoter to reach high expression levels in all cell types (Figure S1A). From 24 to 120 hpf in all districts analyzed, the fluorescence confirmed the cytoplasmic-specific localization of the fusion protein, thus excluding potential nuclear functions (Figure 2I,J; n = 22).

## 3. Discussion

In the proposed work we elucidated key questions concerning the role of *SMPX* mutations in the onset of NSHL by modeling the disease in the lower vertebrate zebrafish *Danio rerio.*

Given that, in patients, hearing loss-causing mutations in *SMPX* are predominantly nonsense, we investigated the role of the gene by means of loss of function assays via gene knockdown. A decrease in the amount of protein was achieved through the injection of two different ATG-targeting morpholinos capable of blocking the translation of *smpx* mRNA.

The first morphological analysis highlighted that Smpx-deficient embryos resulted in being ventrally curved, a specific phenotype that is known to be associated with cilia defects in zebrafish [29,30], thus supporting the idea that Smpx deficiency could lead to ciliogenesis defects in this model.

The effects of *smpx* downregulation were deeply analyzed in the inner ear. Although the functional ablation of the gene did not cause any evident defect in the gross anatomy of the ear, we showed a phenotypic alteration of the apical region of the inner ear hair cells. Indeed, in addition to the apparent lower number of kinocilia, the latter appeared also morphologically altered in that they resulted in being completely straight compared with the curly ones observed in the controls. Moreover, we observed a profound alteration of the kinocilium length and a disarray of the whole stereociliary bundle. As the microtubule-based kinocilium dictates the correct development of the bundle [27,28], we hypothesized that an Smpx deficiency might negatively influence the structure of the kinocilium, thus altering the conformation of the nascent stereociliary bundle. Additionally, as Smpx is localized in the actin-rich CP of the hair cells [4] and participates in the regulation of the dynamics of the cytoskeleton [7], it would be also conceivable that the absence of Smpx might disorganize the actin cytoskeleton of the stereocilia, thus causing the disarray of the sensory bundle. At this juncture, additional experiments are necessary to clarify which one of the hypotheses, or both, takes place during the inner ear hair cell differentiation.

To deeply analyze the role of Smpx in ear functioning, the vital dye FM 4-64, which is known to permeate the non-selective cation channel responsible for mechanotransduction [31], was used as a fast visual readout for potential hearing impairments in zebrafish larvae. Following an injection into the otic capsule, the dye filled the cytoplasm of the control larvae hair cells whereas the hair cells of the Smpx-deficient embryos did not uptake the dye. Consistent with the mechanism of dye permeation through functional (open) MET channels, our data strongly suggested that Smpx-deficient hair cells were no longer able to transduce sound waves. As mentioned above, we previously localized Smpx in the CP of hair cells and not in the stereocilia, nor the kinocilium [4]. In light of this, it is possible to rule out the physical interaction of Smpx with the MET channels, suggesting instead that the alteration of the stereocilia cytoskeleton caused by an Smpx deficiency might destabilize or even impede the physiological interaction of the cytoskeleton itself with other components of the mechanosensory apparatus. Therefore, these data support the hypothesis that the auditory phenotype observed in patients may be due to the lack of mechanotransduction in Smpx-deficient inner ear hair cells, thus providing the first possible explanation for the mechanism underlying hearing loss in humans.

Historically, *SMPX* has always been considered to be an NSHL gene because in human patients no other relevant symptoms have been associated with its mutations [1,2,3,18,19,20,21,22,23,24,25]. Surprisingly, we observed a previously unreported disease phenotype concerning skeletal muscle. Indeed, both slow and fast fibers of Smpx-deficient larvae resulted in being clearly disorganized, less dense and compacted as well as morphologically altered compared with the control ones. Slow and fast muscle fibers are specified during zebrafish embryonic development at two different time points. Specifically, slow and medial fast fibers originate during the so-called first myogenic wave within 24 hpf from the medial and posterior region of the somite, respectively. On the other hand, the external part of the somite, the external dermomyotome, gives rise to the lateral fast muscle fibers at 48 hpf during the second myogenic wave [38]. As *smpx* was expressed in the entire somite during the zebrafish embryonic development [4], it might be involved in both myogenic waves. In line with the diffused disorganization of skeletal muscle fibers observed in Smpx-deficient embryos, the downregulation of the gene is associated with a severe movement phenotype consistent with a diminished muscle function. The muscular developmental impairment reported in this work contrasted with the data by Palmer and colleagues, who stated that *Smpx* knockout mice did not display any overt muscle phenotype [9]. On the contrary, in agreement with our results, it has been recently shown that missense mutations in *SMPX* were associated with the first X-linked recessive form of distal myopathy in humans [35], suggestive of the gene playing a role in proper muscle organization.

## 4. Material and Methods

All of the experiments here described were run on a minimum of three independent batches of embryos. The number (n) of embryos employed is indicated in the main text or in the figure legends.

### 4.1. Zebrafish Husbandry and Maintenance

Zebrafish embryos and larvae of the AB strain were obtained through natural spawning of wild type adult fish raised at 28 °C in the presence of 0.002% Methylene Blue and 0.003% PTU (1-phenyl-2-thiourea) and staged as previously described [39].

Our facility strictly complies with the relevant European (EU Directive 2010/63/EU for animal experiments) and Italian (Legislative Decree No. 26/2014) laws, rules and regulations as also confirmed by the authorization issued by the municipality of Milan (PG 384983/2013). The procedures were carried out in accordance with the relevant guidelines and regulations.

### 4.2. Injections

Injections were carried out on 1- to 2-cell stage embryos adding the rhodamine fluorescent tracer to the solutions.

For the Smpx subcellular localization analysis, a construct bearing an Smpx:GFP fusion ORF was generated. Briefly, a *Bam*HI restriction site was PCR-inserted in place of the *smpx* stop codon (smpx_Forward0: TTGACATTTGTGTCTGACACCA and smpx_Reverse_BamHI: gccGGATCCGTCTTTGGTGACCCATCTGA). The full-length cDNA was digested with *Bam*HI and ligated to the pCS2+ plasmid previously linearized with the same restriction enzyme. The ligation served as a template for a PCR with the an *smpx* forward primer spanning the ATG region and including the endogenous Kozak sequence (smpx_F1: ACTGCACACAATGTCAAAACA) and the M13 reverse universal primer mapping downstream the GFP ORF. The amplicon was then cloned into the pCMV-SC plasmid (Clontech, Mountain View, California, USA) according to the manufacturer’s instructions and 300 pg of the final construct (pCMV-SC_Smpx:GFP) was injected per embryo. The embryos were imaged by a Leica DFC310 FX digital camera and the Leica Application Suite (LAS) software (Leica, Wetzlar, Germany) on a Leica MZ10 F fluorescent stereomicroscope.

To repress the *smpx* mRNA translation, two different ATG-targeting morpholinos (MO1 and MO2) were synthesized (Gene-Tools): 5′-AAGGAAAGTGCTGTTCCCTGGTGTC-3′ (MO1) and 5′-CAGTTGTGTCCACGTTCCTGCTGCT-3′ (MO2). MO1 and MO2 stocks were diluted in Ringer’s solution (116 mM NaCl, 2.9 mM KCl, 1.8 mM CaCl_2_, 5 mM HEPES, pH 7.3) and injected at the concentration of 0.3 and 0.15 pmole per embryo, respectively, in a 4 nL droplet. As a control, 0.3 pmole per embryo of a standard control MO (ctrl-MO) was injected. For the in-vivo test of the specificity of both MOs, 200 pg per embryo of the pCMV-SC_Smpx:GFP construct was co-injected with each one of the *smpx*-MOs or the ctrl-MO, respectively. The presence/absence of the GFP signal was monitored from 24 to 120 hpf. The embryos were imaged by the Leica digital camera DFC310 FX and the Leica Application Suite (LAS) software (Leica) on a Leica MZ10 F fluorescent stereomicroscope.

For the transient gene overexpression assays, *atoh1a*, *smpx* and GFP synthetic mRNAs were transcribed with the mMESSAGE mMACHINE T3 transcription kit (Thermo Fisher) using full-length cDNAs cloned in the pCMV-SC vector (Clontech) as a template. Analogously, the *smpx* synthetic mRNA to rescue the MO-induced effects was transcribed starting from a full-length cDNA cloned in the pCMV-SC vector (Clontech) but lacking the endogenous 5′UTR where the target sequences of the MOs were mapped. The mRNA was co-injected with each one of the 2 MOs separately. The mRNAs were injected at the concentration of 600 pg per embryo in a 4 nL droplet.

### 4.3. RNA Extraction and Quantitative RT-PCR (qRT-PCR)

Total RNAs were extracted from at least 10 embryos per group by using The ReliaPrep™ RNA Tissue Miniprep System (Promega, Madison, WI, USA) according to the manufacturer’s instructions. Six-hundred ng of total RNA was used for the cDNA synthesis using the ImProm-II™ Reverse Transcription System (Promega) and random hexamers (GeneSpin Srl). A quantitative RT-PCR was performed using the CFX Connect Real-Time PCR Detection System (Bio-Rad Laboratories, Inc., Hercules, CA, USA). RT-PCRs were carried out with three technical replicates for each sample in a total volume of 20 μL containing a half volume of SYBR Green Master Mix (GeneSpin Srl, Milan, Italy), 0.2 μM of each primer and an adequate volume of template cDNA. For normalization purposes, the *rpl8* RNA level was tested in parallel with the gene of interest. Melting curves were also assessed for each run.

The experiments were run as three independent replicates and the data were expressed as fold changes in the *smpx*-MO-injected embryos over the controls.

Data were analyzed by using the comparative ΔΔCt method. The statistical analysis was performed in GraphPad Prism 8 by applying a two-tailed *t*-test with Welch’s correction setting of *p* ≤ 0.05 as significant.

### 4.4. Whole-Mount In-Situ Hybridization

*fgf8*, *pax5*, *smyhc1*, *myhz2* and *smpx* antisense riboprobes were transcribed with digoxigenin modified nucleotides (Roche). Whole-mount in-situ hybridizations were carried out as previously described [40] on embryos fixed overnight in 4% paraformaldehyde/PBS (phosphate buffered saline), rinsed with PBS-Tween^®^-20, dehydrated in 100% methanol and stored at −20 °C until processed for WISH. The embryos were imaged by a Leica DFC310 FX digital camera and the Leica Application Suite (LAS) software v4.7 on a Leica MZ10 F stereomicroscope.

### 4.5. Immunofluorescence and Phalloidin Staining

Whole-mount immunofluorescence (IF) assays were performed according to a routine protocol [4]. Fresh embryos were fixed overnight in 4% paraformaldehyde/PBS, washed with PBS/Tween^®^-20/Triton™X-100 and then incubated with 5% BSA (bovine serum albumin, Merck, Darmstadt, Germany) and primary antibodies, anti-acetylated α-tubulin (T7451, Merck), anti-myosin heavy chain (A4.1025, Sigma–Aldrich, St. Louis, MO, USA) and an SPMX-polyclonal antibody (PA3-070, Thermo Fisher); Goat anti-mouse Alexa fluor^TM^ 488 (Thermo Fisher, Waltham, MA, USA) and goat anti-rabbit Alexa fluor^TM^ 555 (Thermo Fisher) were used as fluorescent secondary antibodies. The cell nuclei were visualized by 4′,6-diamidino-2-phenylindole (DAPI). Phalloidin staining was performed on embryos fixed overnight in 4% paraformaldehyde/PBS and subsequently washed with PBS. The samples were stained with a 50 g/mL fluorescent Phalloidin-Atto 550 (19083, Sigma–Aldrich) conjugate solution in PBS for an overnight incubation. All images were acquired by a Nikon A1 laser-scanning confocal microscope (Nikon Instruments Inc.).

For histological sections, the stained embryos were dehydrated, wax embedded and sectioned by a microtome (Leitz 1516) into 7 μm slices. The staining with hematoxylin and eosin was performed according to standard protocols [41]. The images were acquired with a Leica DFC450C digital camera and the Leica Application Suite (LAS) software (Leica) on a Leitz DM RB microscope.

### 4.6. FM 4-64 Dye Microinjection: In Vivo Mechanotransduction Assay

In addition to the conventional mechanism of plasma membrane labelling, FM dyes quickly fill the cytoplasm of mechanosensory hair cells by rapidly entering through open and functional MET channels [34]. One mM of a stock solution was prepared from lyophilized FM^®^ 4-64 dye (Thermo Fisher Scientific) in DMSO. To label inner ear hair cells (IHCs), both 5 dpf morphant and uninjected control live embryos were anesthetized using 1X tricaine in embryo water and laterally oriented on a slide above a bed of 1% agarose. Two nL of a 3 µM FM 4-64 dilution in Ringer’s solution (pH 7.3) was microinjected into the otic capsule [34]. The embryos were immediately subjected to confocal imaging.

### 4.7. Scanning Electron Microscopy (SEM)

Seventy-two hpf embryos were fixed for 24 h with 2% glutaraldehyde in a 0.1 M sodium cacodylate buffer (pH 7.2). After fixation, the samples were extensively washed with a 0.1 M sodium cacodylate buffer (pH 7.2) and post-fixed with 2% osmium tetroxide in distilled water. The samples were then dehydrated with an ethanol series (50%, 70%, 90% and 100%) and further processed with a critical point dryer (Balzers Union, Liechtenstein). The dried samples were mounted on standard aluminum pin stubs and coated with a thin layer of gold before imaging. The images were acquired with a Zeiss LEO 1430 SEM (Carl Zeiss Jena GmbH, Germany). The SEM images were acquired with an accelerating voltage ranging from 10 to 20 kV, a probe current of 80 µA and a working distance ranging from 10.0 to 15.0 mm at different magnifications from several areas of the ear.

### 4.8. Touch-Evoked Response Assay

The skeletal muscle function in Smpx-deficient embryos was assessed by a touch-evoked response assay [42]. Forty-eight hpf embryos were transferred singularly to a Petri dish (Ø = 4 cm) filled with fish water and left 1 min for acclimation. A mechanosensory stimulus was then delivered by gently touching the embryo on the side of the head with a blunt needle. The embryos were imaged by a Leica DFC310 FX digital camera and the Leica Application Suite (LAS) software on a Leica MZ10 F stereomicroscope and videos were acquired using the Apowersoft online screen recorder (Apowersoft). The videos were analyzed with Wondershare Filmora 9 (Wondershare Technology Co., Ltd., Lhasa, China) to extract single frames at the desired timepoints.

## 5. Conclusions

In summary, our work provides the functional evidence that strongly supports previous data connecting *SMPX* mutations and hearing loss in humans [1,2,3,18,19,20,21,22,23,24,25]. Indeed, *smpx* downregulation in zebrafish embryos resulted in profound structural and functional alterations of the larval inner ear, also raising the possibility that *SMPX*-linked hearing loss might represent a developmental defect, as suggested by some cases of pre-lingual hearing loss [23] and not just the result of the lack of a protein involved in preserving the hair cells from mechanical stress [43]. Here we provided a new model to study the precise cellular and molecular mechanisms by which mutations in *SMPX* cause hearing disorders, paving the way towards the identification of therapeutic interventions. Our results also unveil potential new roles for this gene, as *smpx* was heavily involved in the proper muscle development of zebrafish. Interestingly, this piece of evidence agrees with the recently reported association between *SMPX* mutations and distal myopathy [35]. Therefore, more in-depth analyses in search for possible correlations between *SMPX* mutations and muscular disorders in humans are needed, thus potentially turning this non-syndromic hearing loss-associated gene into the genetic cause of dysfunctions characterized by more than one symptom, making *SMPX* a novel syndromic gene.

## Data Availability

The data presented in this study are available on request from the corresponding author.

## Supplementary Materials

The following are available online at https://www.mdpi.com/article/10.3390/ijms22126497/s1.
