# Supplementary material for: Inner Ear and Muscle Developmental Defects in Smpx-Deficient Zebrafish Embryos
Source: Int J Mol Sci. 2021 Jun 17;22(12):6497. doi: 10.3390/ijms22126497 (PMC8235540; doi:10.3390/ijms22126497)
Supplement: Supplementary file 1 [file ijms-22-06497-s001.zip › ijms-1245595-supplementary.pdf]

International Journal of *Molecular Sciences*

MDPI

**SUPPLEMENTARY MATERIALS**

Article

# Inner Ear and Muscle Developmental Defects in Smpx-Deficient Zebrafish Embryos

**Anna Ghilardi [1], Alberto Diana [1], Renato Bacchetta [2], Nadia Santo [3], Miriam Ascagni [3], Laura Prosperi [1] and Luca Del Giacco [1,*]**

[1] Department of Biosciences, Università degli Studi di Milano, Via Celoria 26, 20133 Milan, Italy; anna.ghilardi@unimi.it (A.G.); alberto.diana@unimi.it (A.D.); lau.prosperi@gmail.com (L.P.)
[2] Department of Environmental Science and Policy, Università degli Studi di Milano, 20133 Milan, Italy; renato.bacchetta@unimi.it
[3] Unitech NOLIMITS, Università degli Studi di Milano, 20133 Milan, Italy; nadia.santo@unimi.it (N.S.); miriam.ascagni@unimi.it (M.A.)
* Correspondence: luca.delgiacco@unimi.it

**Figure S1:** *smpx*-MOs reduce smpx mRNA translation.

**Figure S2:** Lack of Smpx does not affect the gross morphology of the otic vesicle.

**Figure S3:** Smpx-deficiency is associated to profound alterations of the ciliary bundle.

**Figure S4:** *atoh1* overexpression does not affect the distribution of *smpx* specific signal in the inner ear of 48 hpf larvae.

**Figure S5:** Smpx is critical for the proper organization of the larvae muscle fibers.

**Figure S6:** Smpx deficiency impairs larval muscle functioning.

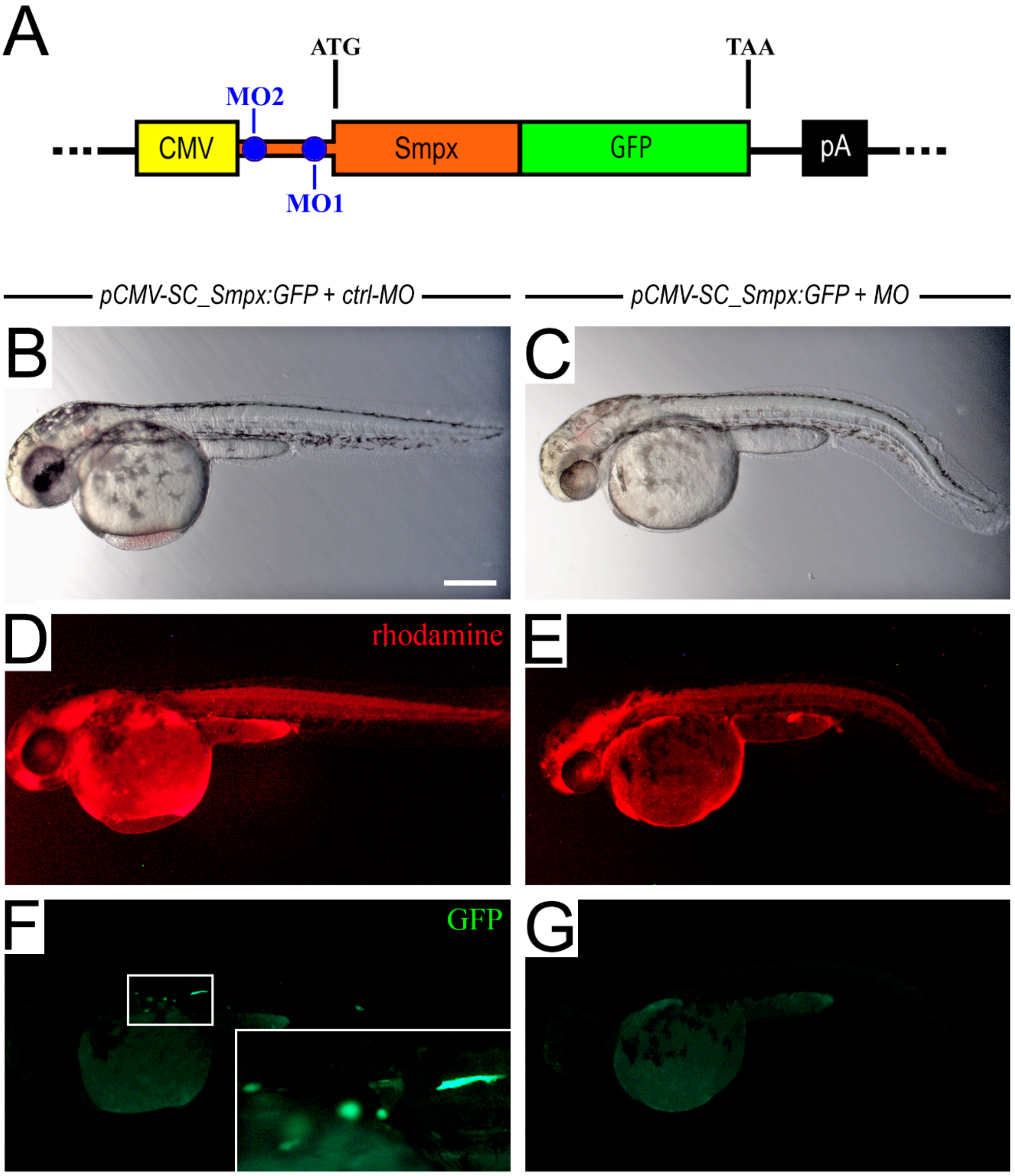


**Figure S1.** smpx-MOs reduce smpx mRNA translation. (**A**) the pCMV-SC_Smpx:GFP is used as a sensor to test the activity of the two *smpx*-MOs *in vivo*. The blue circles indicate the regions of the mRNA targeted by the MOs (MO1 and MO2). (**B**,**C**) representative brightfield images of pCMV-SC_Smpx:GFP (B) and pCMV-SC_Smpx:GFP/MOs (C) injected embryos. (**D**,**E**) fluorescent signal of the rhodamine dye co-injected as a tracer. (**F**,**G**) GFP-positive cells (F and inset) are visible following coinjection of the sensor and the standard control MO (ctrl-MO). The complete absence of fusion protein expression when the sensor is coinjected with either one of the two *smpx*-MOs (G) confirms the ability of both MO1 and MO2 in blocking mRNA translation. Scale bar represents 110 μm.

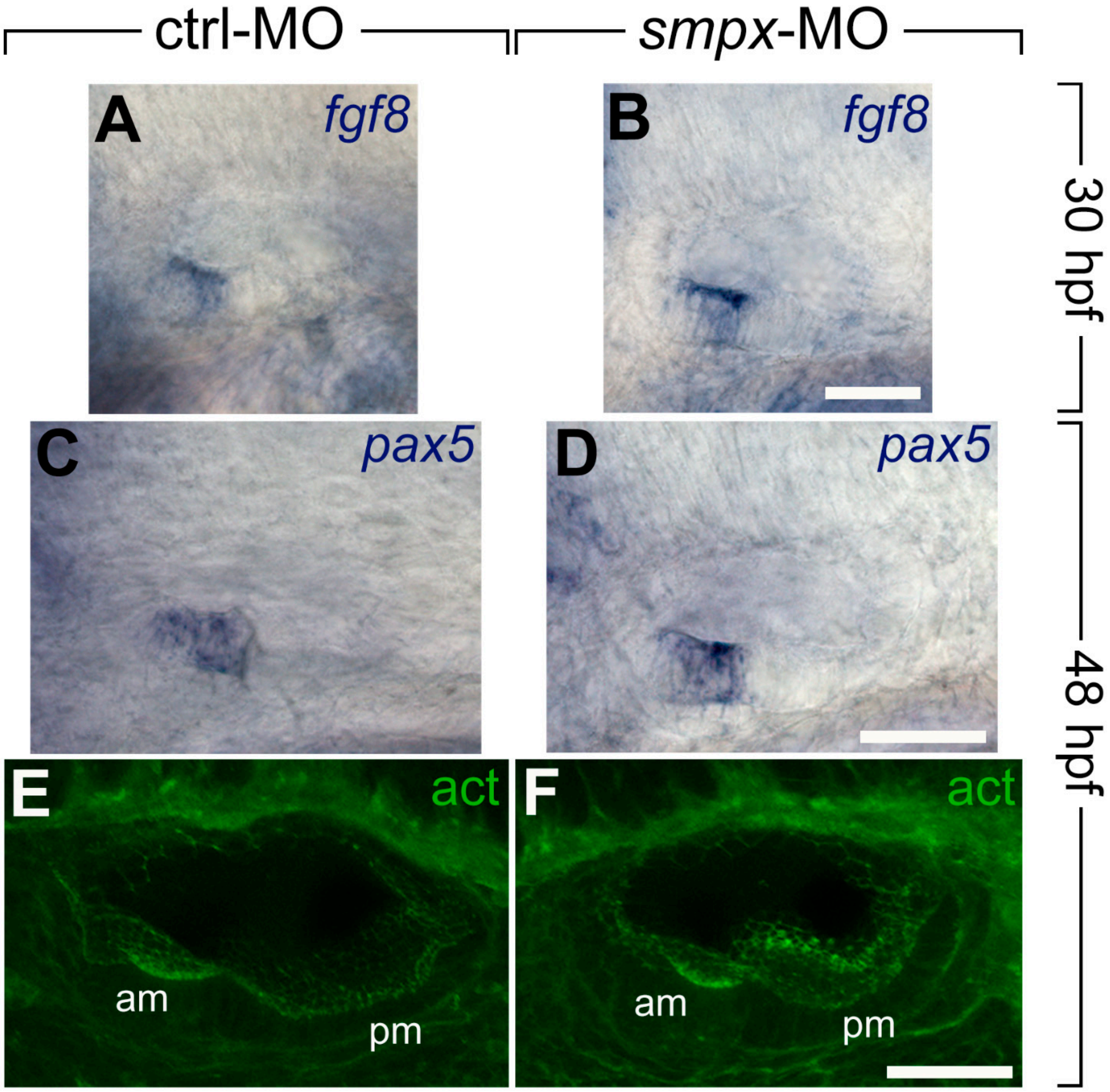


**Figure S2.** Lack of Smpx does not affect the gross morphology of the otic vesicle. (**A-D**) whole mount *in-situ* hybridizations were carried out using *fgf8* (A,B) and *pax5* (C,D) specific riboprobes. (**E-F**) actin filaments of the cytoskeleton of the ear cells stained with phalloidin to highlight the anterior (am) and posterior (pm) maculae.

The expression of the two ear specific markers, as well the actin staining, do not reveal any visible phenotypic alteration of the structure.

Scale bars = 50 µm.

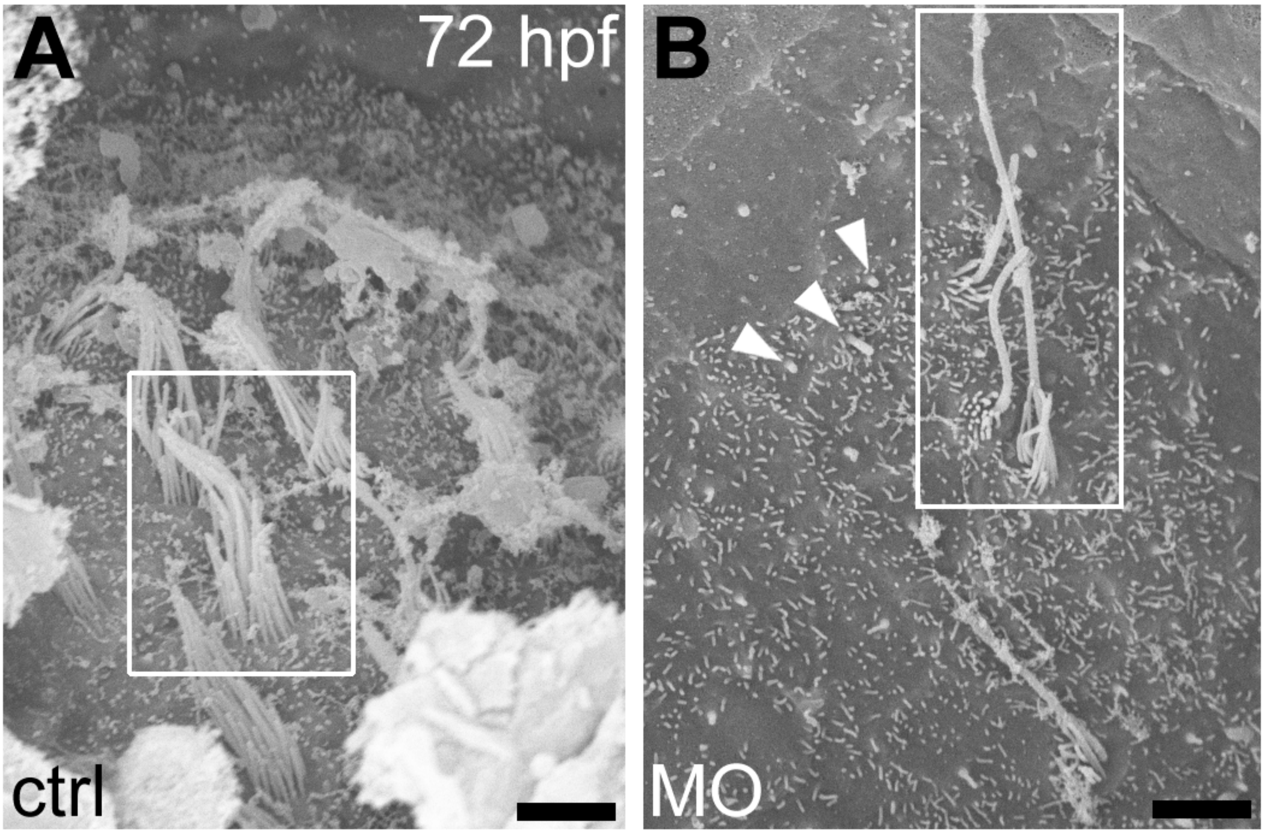


**Figure S3.** Smpx-deficiency is associated to profound alterations of the ciliary bundle. (**A,B**) Representative SEM images of ciliary bundles in control larvae (A) and Smpx-morphants (B) at 72 hpf. The very few morphant kinocilia (B) are misdeveloped (some hyperdeveloped – white rectangle - some others very short – white arrowheads) and surrounded by numerous, extremely small stereocilia which are dispersed instead of being organized in the typical bundle embracing the kinocilium (A). The white rectangles in A and B depict the regions showed in figure 1G and 1H, respectively.

Scale bars = 2 μm.

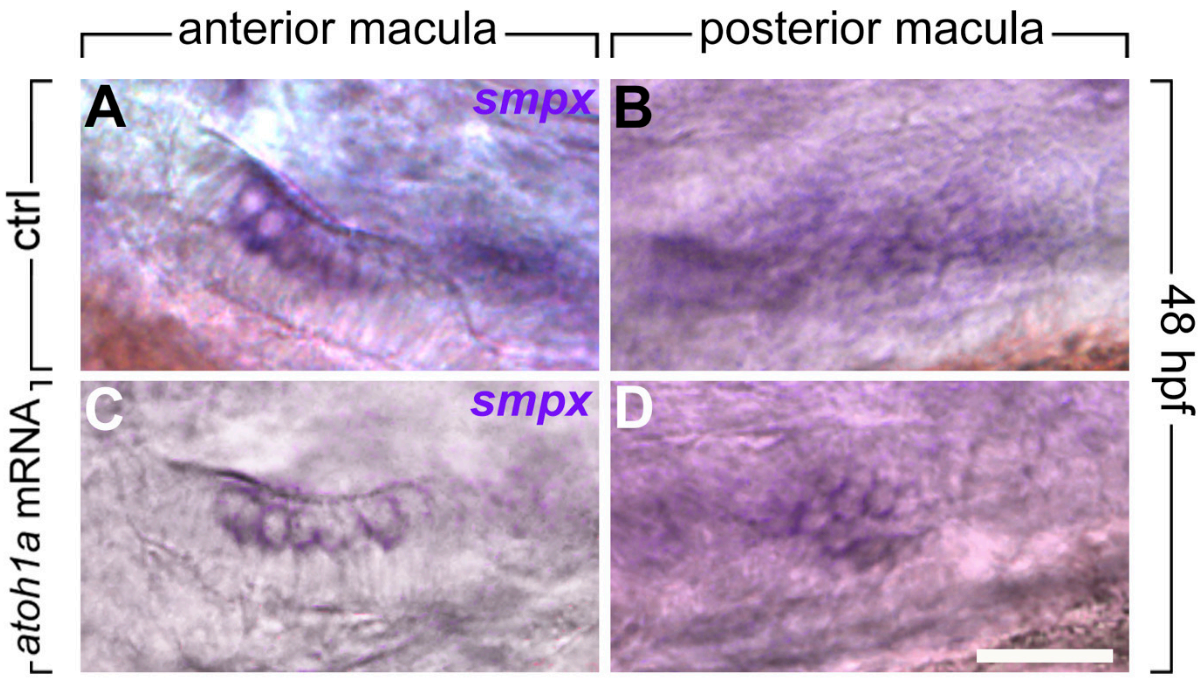


**Figure S4.** *atoh1* overexpression does not affect the distribution of *smpx* specific signal in the inner ear of 48 hpf larvae. (**A-D**) whole mount *in-situ* hybridizations carried out with the *smpx* specific riboprobe on control embryos (A,B), injected with GFP-encoding mRNA, and embryos injected with the *atoh1a* transcript (C,D). Anterior (A,C) and posterior (B,D) maculae do not show any alteration in the *smpx* expression pattern following *atoh1a* upregulation.

Scale bars = 50 µm.

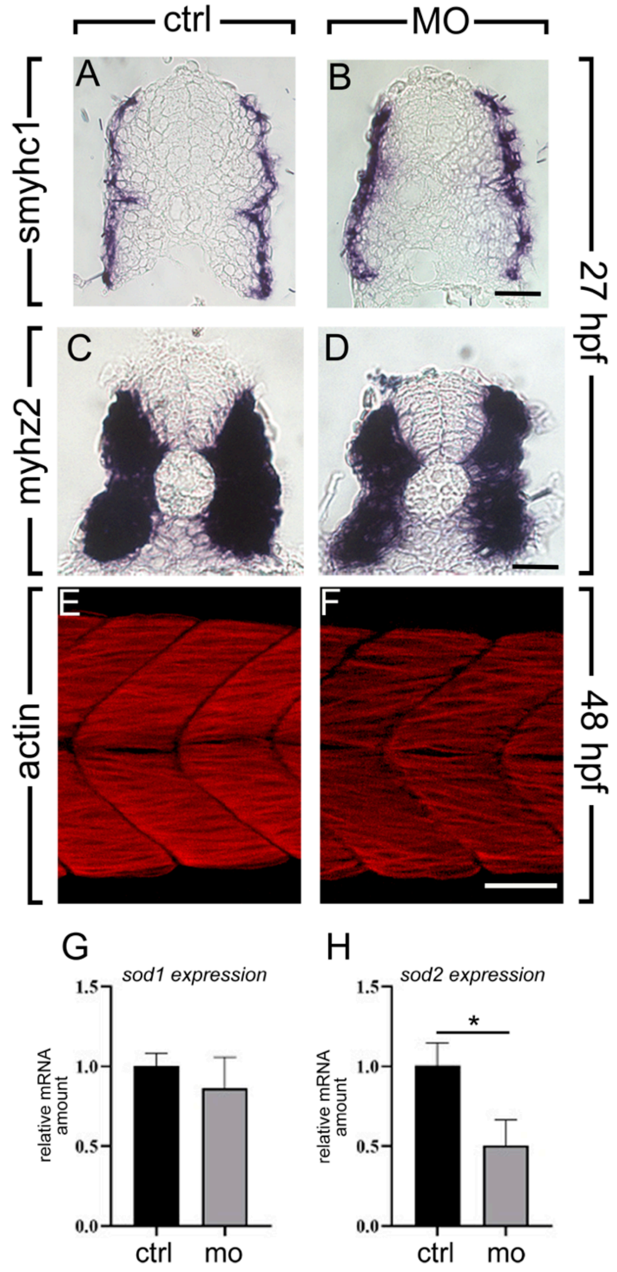


**Figure S5.** Smpx is critical for the proper organization of the larvae muscle fibers. (**A-D**) histological sections of whole embryos hybridized with the *smyhc1* (A,B) and *myhz2* (C,D) specific riboprobes, painting the slow and fast muscle fibers, respectively. Smpx downregulation does not alter the slow/fast fibers balance between controls (ctrl), injected with the standard morpholino (A,C), and Smpx-deficient embryos (B,D). Scale bars = 15 μm. (**E,F**) actin filaments of the muscle fibers of the trunk stained with phalloidin in controls (ctrl), injected with the standard morpholino (E), and Smpx-deficient embryos (F). Scale bar = 200 μm. **G,H**: qRT-PCR analysis of *sod1* (G) and *sod2* (H) expression showing the significant reduction of the amount of the sole *sod2*-specific mRNA. Data are expressed as fold change in *smpx*-MO-injected embryos (MO) over controls (ctrl). Experiments were run as three independent replicates (**P* $\leqslant$ 0.05).

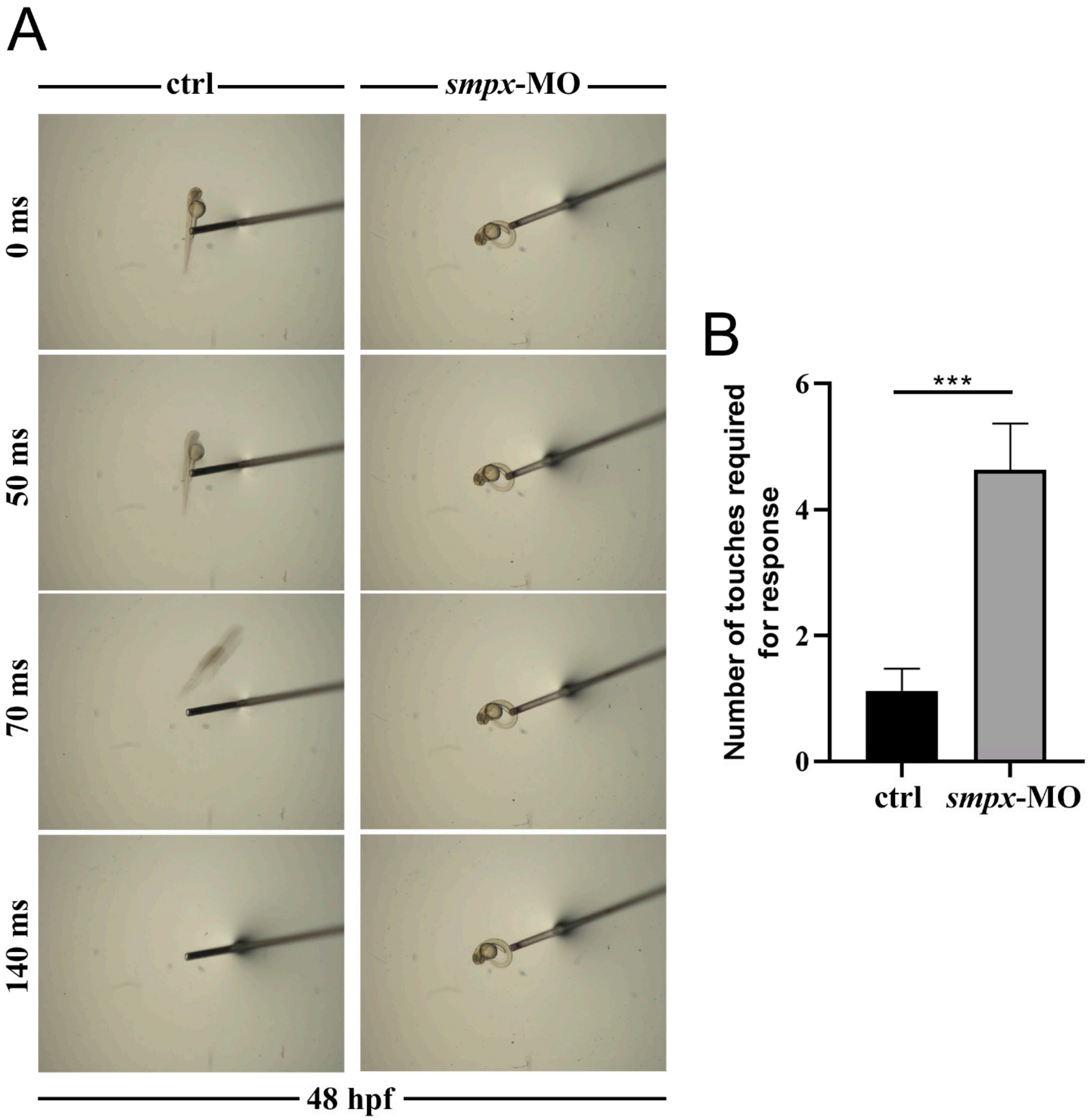


**Figure S6.** Smpx deficiency impairs larval muscle functioning. (**A**) selected images from videos recorded immediately after touching control (ctrl) and Smpx-deficient (*smpx*-MO) embryos. The typical escape response behavior, displayed by controls (left column) and showed in milliseconds (ms), is dramatically decreased in Smpx-morphants (right column). (**B**) quantification of the number of stimuli required to induce the escape response. Eight embryos for each group were used. For quantification purposes, only the first 5 touches were considered, according to Mastrodonato and colleagues (Mastrodonato et al., 2019). P-values were obtained by applying the Mann-Whitney statistical test in GraphPad Prism 8 (***$P \leqslant 0.001$).
